# Lower Motor Neuron Facial Palsy Due to Facial Colliculus Syndrome

**DOI:** 10.7759/cureus.25053

**Published:** 2022-05-16

**Authors:** Yi-En C Seah

**Affiliations:** 1 Accident and Emergency Department, Changi General Hospital, Singapore, SGP

**Keywords:** vertigo, bell`s palsy, facial colliculus syndrome, facial colliculus infarct, facial palsy

## Abstract

In patients presenting to the Emergency Department (ED) with acute onset facial asymmetry, decision for disposition is usually based on whether it is an upper (UMN) or lower motor neuron (LMN) cranial nerve 7^th^ (CN7) palsy. In my institution, patients with UMN CN7 palsy would require admission for further investigations to look for central causes. Those who have an isolated LMN facial nerve palsy can be managed as outpatients.

A 36-year-old gentleman presented to the ED with acute vertigo and right facial weakness. He had no known cardiac risk factors. His vital signs on presentation were: Temperature 36.6℃, blood pressure 142/68mmHg, pulse rate 92/min, and oxygen saturation level (SpO2) 100% on room air. Initial neurological examination revealed a right LMN CN7 palsy without any other cranial nerve, cerebellar, or pyramidal deficits. He was given symptomatic treatment for vertigo without relief. Repeat examination subsequently showed a right conjugate gaze palsy with gaze-evoked nystagmus. There was no limb weakness or numbness. Gait was noted to be unsteady with a broad-based stance and truncal ataxia. Magnetic resonance imaging (MRI) of his brain subsequently showed an infarct affecting the right facial colliculus in the dorsal pons.

In my department, this was the first case of a young patient with a stroke presenting with LMN CN7 palsy. He was initially treated for a possible peripheral cause of his vertigo as he had a history of vestibular neuronitis, but without symptomatic improvement. Patients with neurological symptoms (e.g. vertigo) not resolving with initial treatment should prompt consideration for repeat neurological examination because the patient may have evolving neurological signs, as well as consider the potential for initial anchoring/cognitive bias. In this case, the gaze palsy and cerebellar signs were only noted on subsequent examination. Presence of LMN CN7 palsy with other associated neurological signs (including other cranial nerve palsies) would warrant further imaging to look for more sinister intracranial causes, including cerebral infarcts or space-occupying lesions.

This case serves to remind medical practitioners to strongly consider a central cause (e.g. stroke) for patients presenting with an LMN facial palsy, even in young patients in the absence of other vascular risk factors, especially when other neurological symptoms and signs are present.

## Introduction

The Emergency Department (ED) is frequently the first place any patient with neurological symptoms or signs would present to. In the assessment of facial palsy, the commonest approach would be to divide it into an upper motor neuron (UMN) or lower motor neuron (LMN) lesion. A UMN pattern of weakness affecting the facial nerve would present with weakness/paralysis of the contralateral mid to lower half of the face due to dual innervation of the dorsal aspect of the facial motor nucleus from the motor cortex. Complete paralysis of one-half of the face would constitute a LMN pattern of weakness (ipsilateral). Some studies put the incidence of facial nerve palsy between 20 to 32 per 100,000 person-years [1,2]. A survey done in the United States (US) showed the five most common etiologies as idiopathic, infectious, neoplastic, neurologic, and traumatic [3], the most common of which is Bell’s palsy. In some studies, it accounted for over 60% of peripheral facial palsies [1,4]. Here is a case of a young gentleman who presented with right LMN CN7 palsy and vertigo, secondary to an infarct in the right facial colliculus.

This article was previously presented as a poster at the Xth Mediterranean Emergency Medicine Congress on 22-25 September 2019 and has been updated.

## Case presentation

Mr MS was a 36-year-old Indian male who presented to the ED of a tertiary teaching hospital with sudden onset of vertiginous dizziness associated with vomiting. His vital signs on presentation were: Temperature 36.6℃, blood pressure 142/68mmHg, pulse rate 92/min, and oxygen saturation level (SpO2) 100% on room air. He also complained of right-sided facial weakness of one day’s duration with blocked ear sensation. He had a history of vestibular neuronitis and recurrent vestibulopathy, for which previous work up with Computed Tomography (CT) and Magnetic Resonance Imaging (MRI) of his brain were unremarkable. He complained of difficulty walking due to the giddiness but had no weakness or numbness affecting his arms or legs. He also did not complain of slurred speech. He had taken cinnarizine without improvement. He was an occasional smoker of two to three pack years but had no other known cardiovascular risk factors. He also had no known family history of stroke.

On initial examination, only an isolated right LMN CN7 palsy was detected. He was alert and able to sit up without any evidence of truncal ataxia, or cerebellar signs. Hearing was intact. Sensation over his face was intact bilaterally. Otoscopy was unremarkable without any vesicles in the ears noted. There was no pronator drift, no dysmetria, and no weakness or numbness of all limbs. He was given an intramuscular (IM) injection of prochlorperazine without relief. In view of unresolving symptoms, a repeat neurological examination was performed. He was then noted to have a conjugate gaze palsy to the right side, associated with gaze-evoked horizontal nystagmus. Truncal ataxia was also noted on standing from the chair and gait was unsteady with a broad-based stance. These abnormal neurological findings were not elicited during the initial examination. 

**Figure 1:**
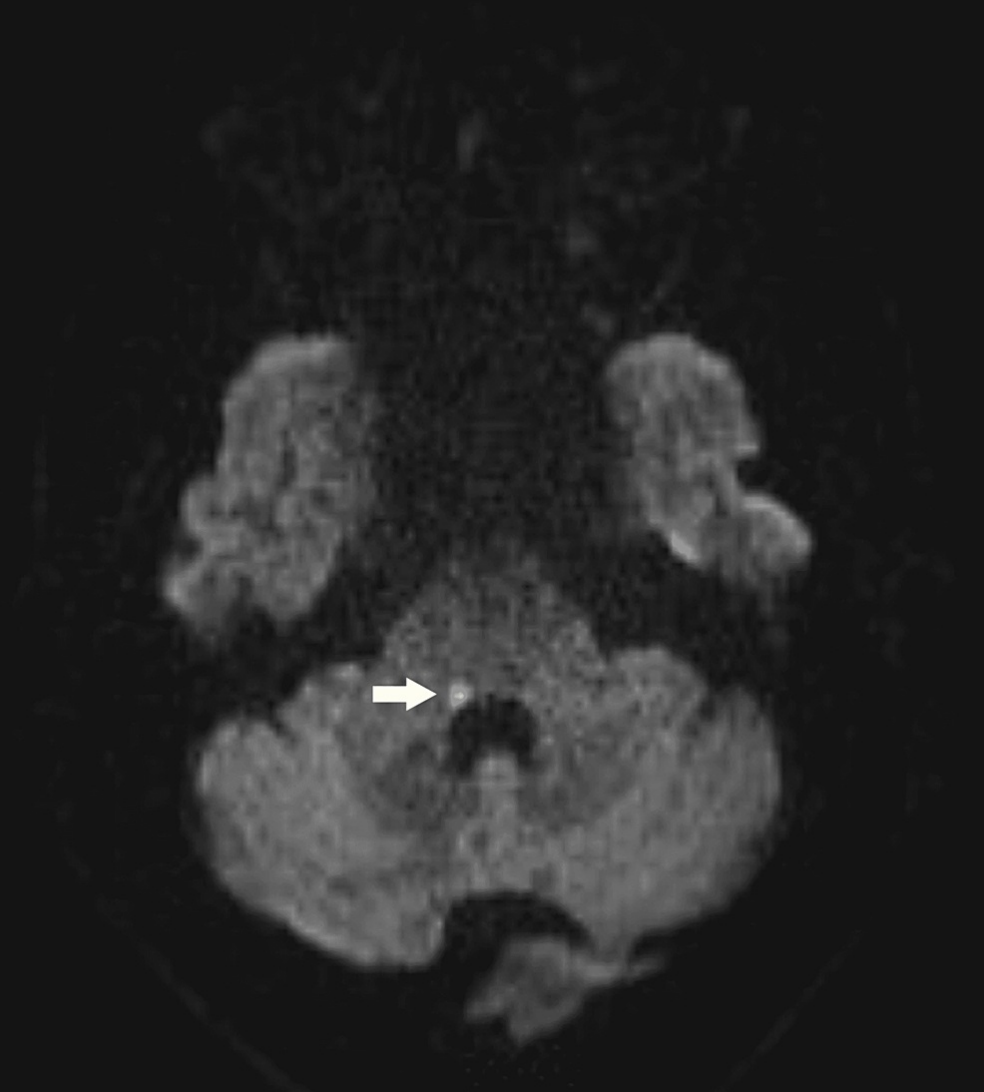
MRI Brain DWI sequence with arrow showing an acute infarct in the right facial colliculus

An immediate CT brain done did not show any large territorial infarct or intracranial hemorrhage. He was admitted to the hospital and MRI brain subsequently showed an acute infarct affecting the right facial colliculus in the dorsal pons shown in Figure 1 below. 

On further workup for cardiovascular risk factors, Mr MS was diagnosed with dyslipidemia (total cholesterol 5.87mmol/L, LDL cholesterol 4.53mmol/L, HDL cholesterol 1.20mmol/L, and triglycerides 1.31mmol/L) and chronic renal impairment, with creatinine of 185µmol/L. Ultrasound kidneys showed increased renal parenchymal echogenicity suggestive of underlying renal parenchymal disease. There was no evidence of renal artery stenosis and no carotid artery stenosis. He was started on daily aspirin 100mg and atorvastatin 40mg every night.

## Discussion

**Figure 2:**
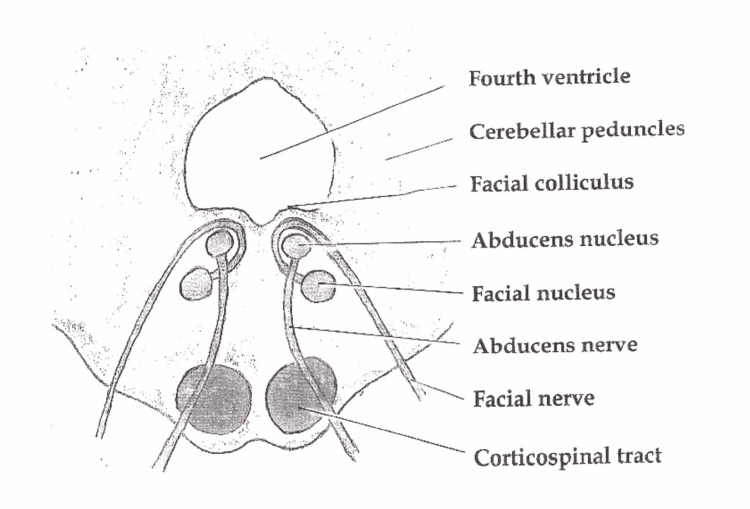
Pons section showing location of facial colliculus Source: https://commons.wikimedia.org/wiki/File:Pons_section_at_facial_colliculus.png Original uploader: btarski

Fisher originally described in 1967 the one and a half syndrome which involves a conjugate lateral gaze palsy with an internuclear ophthalmoplegia [5]. Since then, there have been variations of the one and a half syndrome described, including the eight and a half syndrome [6,7] which is one and a half syndrome with LMN CN7 palsy, as well as eight syndrome [8] which comprises conjugate gaze palsy and LMN CN7 facial palsy. The facial colliculus is an elevation on the floor of the fourth ventricle located in the dorsal pons. It houses the abducens nucleus and facial motor fibers [9] and is formed by the fibres of the facial nerve arching backwards around the abducens nerve nucleus. Figure 2 below shows a cross-section of the pons with location of the facial colliculus [10].

Hence, a lesion affecting the facial colliculus can affect any (or all) of the following structures: Facial nerve fibres at the genu (post nuclear) (CN7), abducens nucleus (CN6), medial longitudinal fasiculus (MLF). In our patient, clinically he had features of eight syndrome which suggest involvement of the right facial nucleus (right LMN CN7 palsy), abducens nucleus and MLF (leading to the right congujate gaze palsy). In addition, his vertigo could be attributed to disruption of the vestibular ocular reflexes that also run in the MLF [11].

The approach to CN7 palsy is dependent on detailed examination, determining if it is an upper or lower neuron paraesis, and specifically looking out for other associated cranial nerve lesions and findings of other long tract signs. Whilst being a very common cause of LMN CN7 palsy, Bell’s palsy is idiopathic and remains a diagnosis of exclusion to this day [4]. Mr MS is young with no known cardiac risk factors at presentation. He had a history of vestibular neuronitis and recurrent vestibulopathy, which might have led to anchoring/cognitive bias by the initial attending clinician who treated him symptomatically as there were no other clinical findings on initial examination elicited to suggest other etiologies. However, when symptomatic treatment did not relieve his vertigo, a repeat neurological examination was performed which revealed a new ipsilateral conjugate gaze palsy as well as truncal ataxia and unsteady gait. This underscores the importance of serial neurological assessments in patients with persistent neurological symptoms. Further workup subsequently revealed dyslipidemia, which might be a contributing risk factor for his stroke. He was also found to have chronic kidney disease, which suggests possible underlying microvascular disease.

## Conclusions

While the features of facial colliculus syndrome can be characteristic once identified, however, neuro-localization based on physical examination may be challenging if the patient does not manifest all the symptoms at presentation. Furthermore, facial colliculus syndrome is not a commonly encountered neuro-ophthalmologic syndrome and emergency physicians or rotating doctors not specializing in neurology may not be familiar with its constellation of signs and symptoms.

This case of Mr MS serves to remind us that an accurate and detailed neurological examination needs to be performed in all patients presenting with neurological symptoms and signs, with serial examinations carried out after a period of treatment and observation to monitor for progression of signs. A central cause should always be considered in patients who are found to have LMN CN7 palsy in addition to other neurological symptoms and signs as Bell’s palsy remains to this day a diagnosis of exclusion. This is so even in young patients without known cardiovascular risk factors as many of the cardiovascular risk factors are only picked up if active screening is done. One should also always be cognizant of the possibility of anchoring/cognitive bias which may cloud initial assessment and if required, to reassess frequently and consider other differential diagnoses should the patient not improve with initial treatment.
